# Supplementary material for: Substrate recognition mechanism of the endoplasmic reticulum-associated ubiquitin ligase Doa10
Source: Nat Commun. 2024 Mar 11;15:2182. doi: 10.1038/s41467-024-46409-2 (PMC10928120; doi:10.1038/s41467-024-46409-2)
Supplement: Supplementary file 1 — Supplementary Information [file 41467_2024_46409_MOESM1_ESM.pdf]

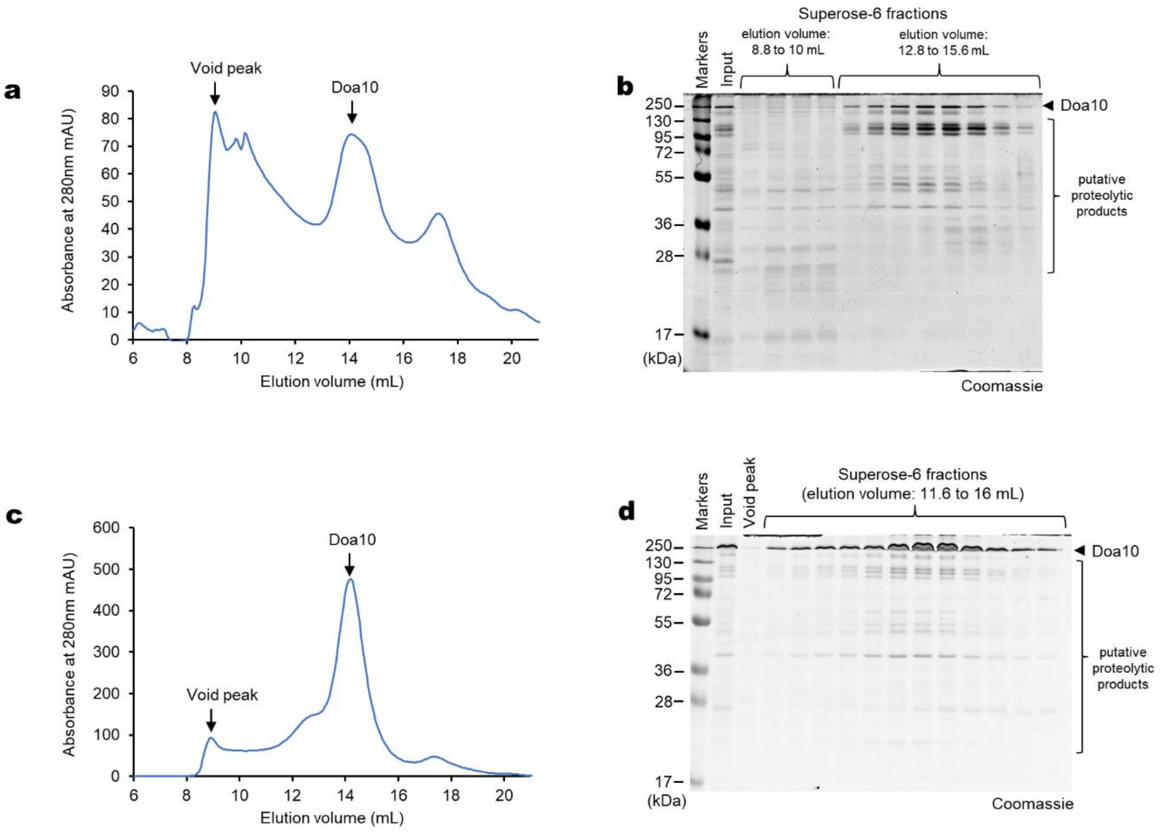

Supplementary Figure 1. Purification of Doa10 from *S. cerevisiae*.
a, Superose-6-increase size-exclusion chromatography (SEC) elution profile of affinity-purified endogenous Doa10 (GFP-tagged Doa10). **b**, Coomassie-stained SDS gel of Superose 6 fractions shown in **a**. **c** and **d**, As in **a** and **b**, but Doa10 was overexpressed by replacing the endogenous promoter with a *GAL1* promoter.

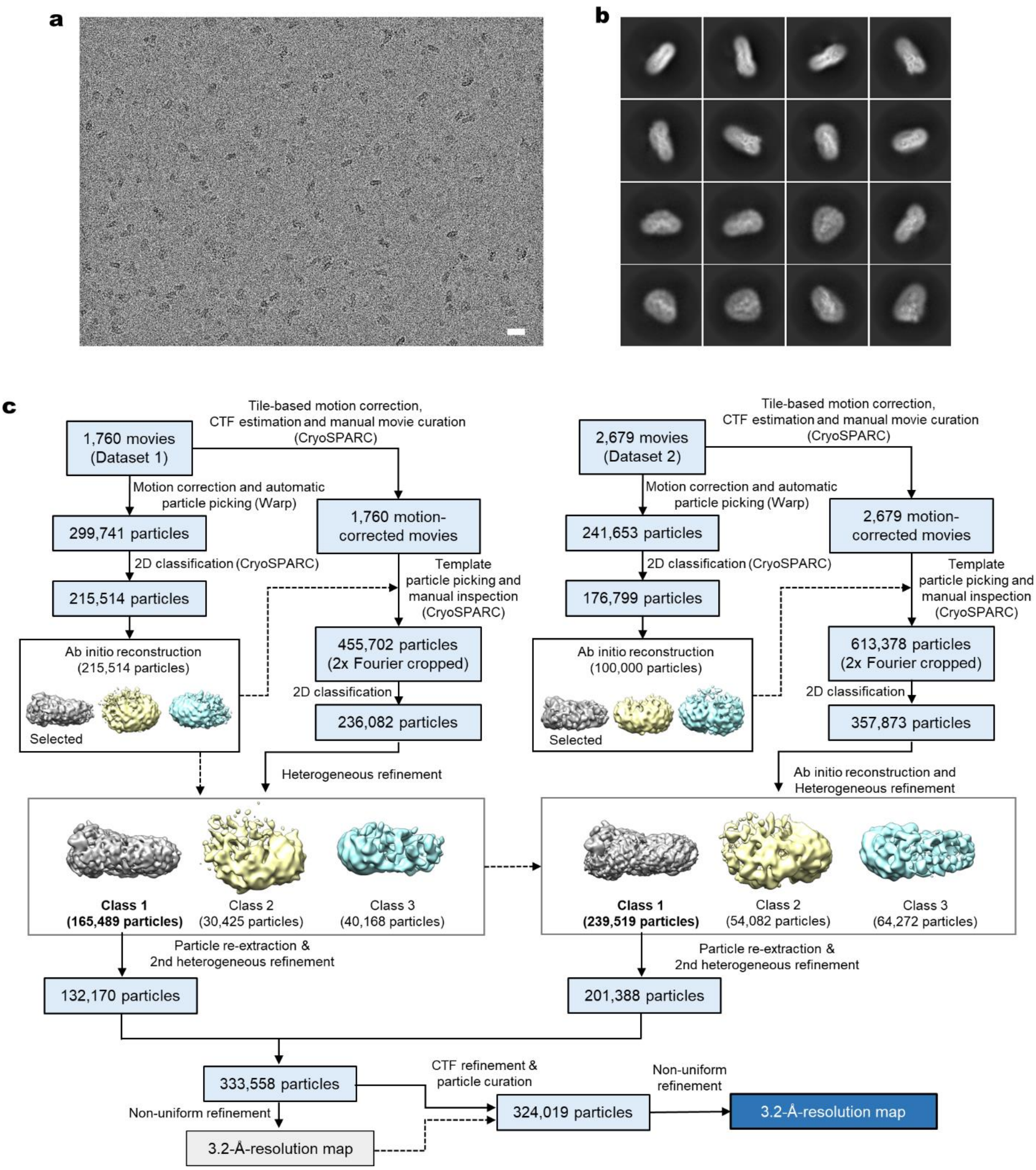

Supplementary Figure 2. Cryo-EM analysis of Doa10.
a, A representative micrograph (cropped) image of Doa10 particles. Scale bar, 200 Å. **b**, Representative 2D class averages. **c**, Schematic diagram for the cryo-EM image analysis procedure.

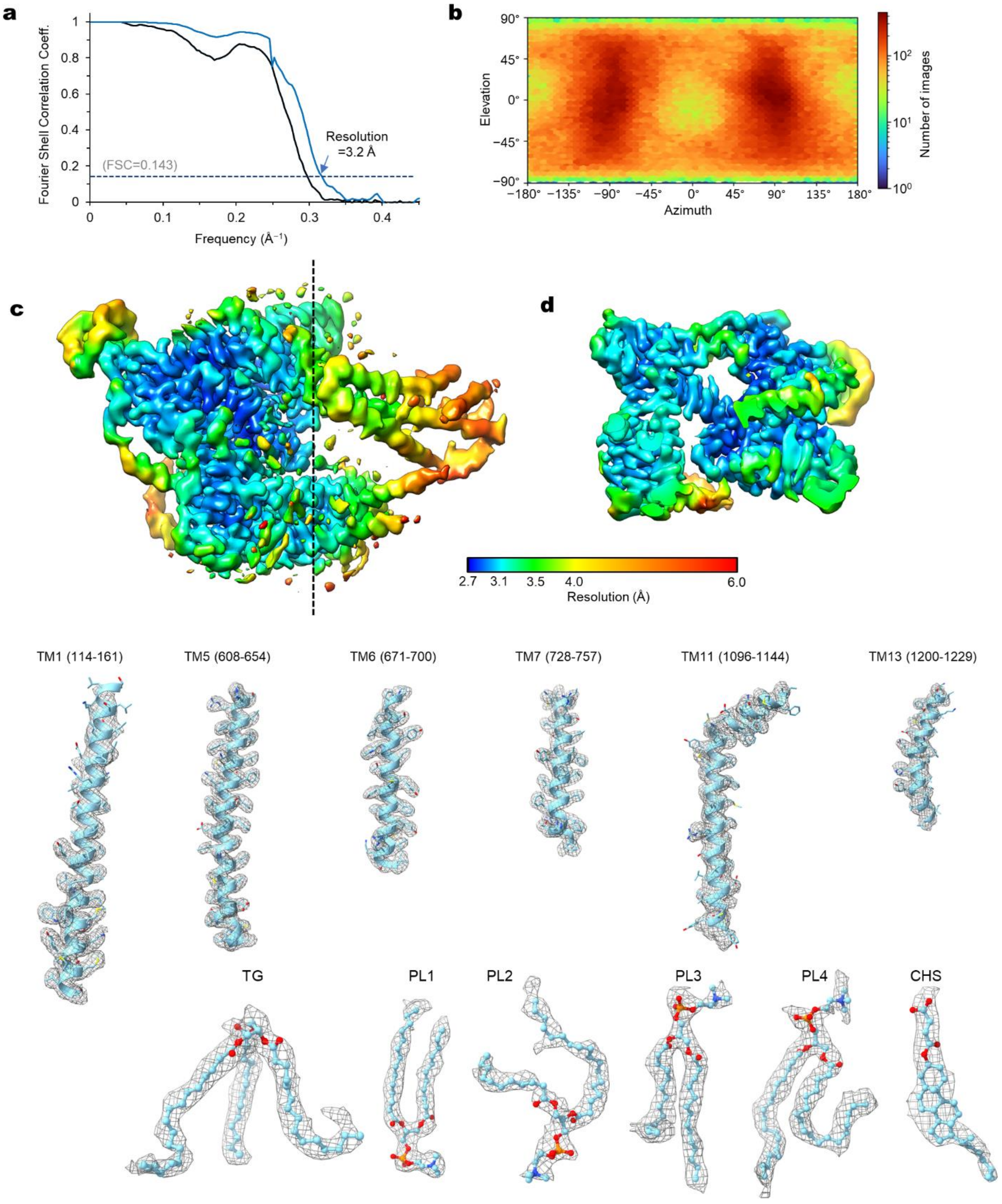

Supplementary Figure 3. Quality of the cryo-EM map of Doa10.
a, Fourier shell correlation (FSC) between the two half maps of the final 3D reconstruction. Blue, tight mask and corrected for masking; black, spherical mask. **b**, Distribution of particle orientation. **c** and **d**, Local resolution map (isosurface is an unsharpened map). The dash line indicates the cutaway plane for the view shown in **d**. **e**, Densities (gray mesh) and models of TM segments and ordered lipids in the Doa10 cryo-EM structure.

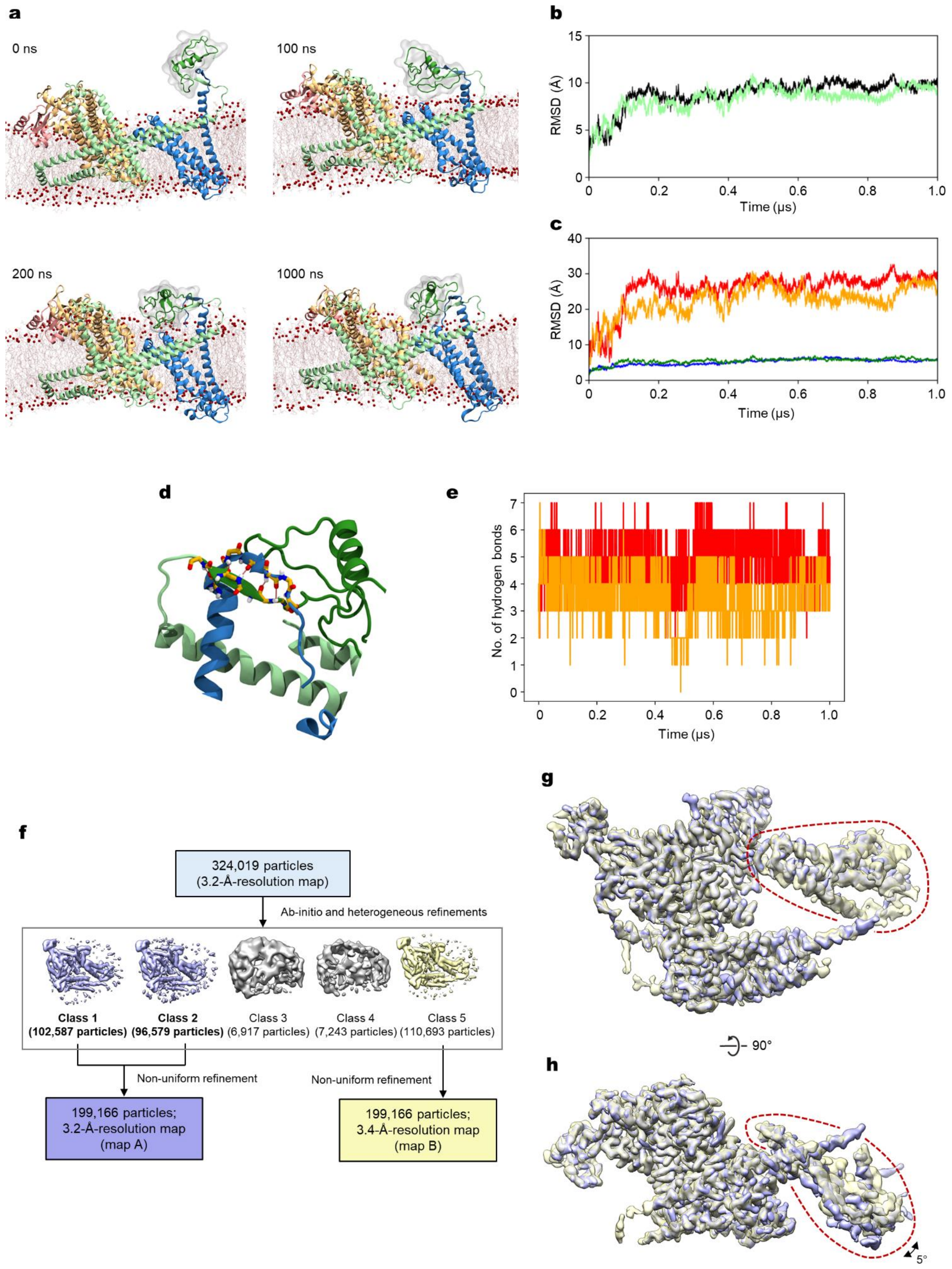

Supplementary Figure 4. Conformational flexibility of the RING-CH domain and CTD of Doa10.
(see next page for legend)

Supplementary Figure 4. Conformational flexibility of the RING-CH domain and CTD of Doa10.
a, Snapshots of MD simulations of wild-type Doa10 in a model membrane. Residues in the simulations include those in the hybrid atomic model detailed in the Methods, specifically residues 30 to 240, 463 to 1052, and 1095 to 1318. The RING-CH domain is shown as a green ribbon and gray semi-transparent surface. **b**, Root-mean-square deviation (RMSD) using the Cα atoms for the two replicas of the Doa10 MD simulations, where alignment is made to the initial structure. **c**, As in **b**, but additionally showing RMSD calculated for the RING-CH domain (red and orange; amino acid residues 30 to 118) or the transmembrane domain (blue and green; amino acid residues 119 to 240, 463 to 1052, and 1095 to 1318) for each replica, without performing additional alignment. **d**, Snapshot of the MD simulations highlighting the two-strand β-sheet formed between the RING-CH and CTE of Doa10. **e**, The number of hydrogen bonds between the RING-CH and CTE over the duration of the MD simulations. Red and orange traces represent the two independent replicas. **f**, Additional classification of the Doa10 cryo-EM dataset to analyze the conformational heterogeneity of CTD. **g** and **h**, The two maps obtained from the procedure in **f** are overlaid. Shown are top (cytosolic; panel **g**) and side (panel **h**) views. CTD is indicated by red dashed lines.

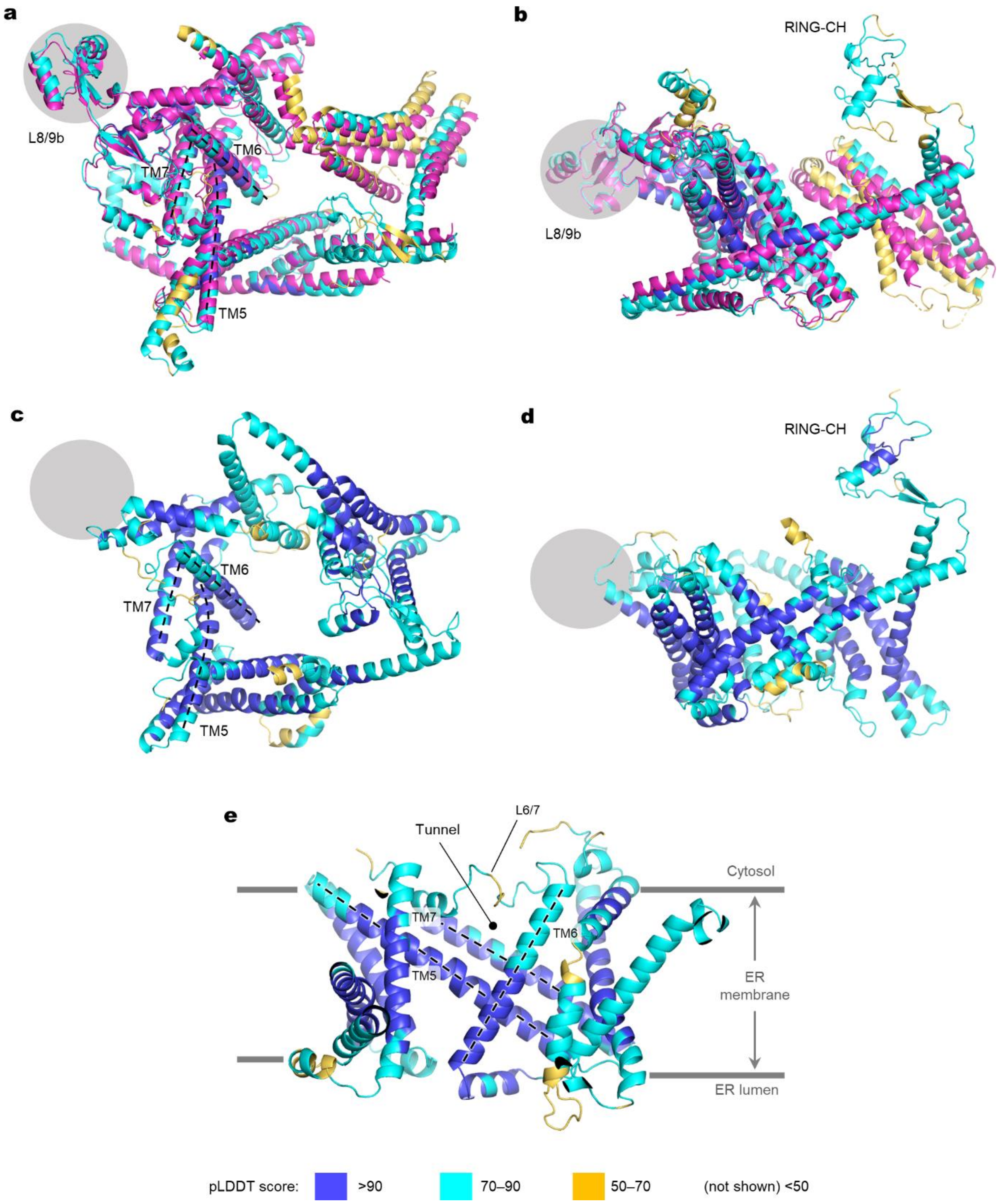

Supplementary Figure 5. AlphaFold2 models of yeast Doa10 and human MARCHF6.
a and **b**, Comparison between the cryo-EM structure (magenta) and AlphaFold2 model (other colors) of yeast Doa10. The AlphaFold2 model is colored according to the pLDDT score (blue, >90; cyan, 70 to 90; yellow, 50 to 70). Parts below less than a pLDDT score below 50 were not shown. Shown are top (cytosolic; panel **a**) and side (panel **b**) views. The L8/9b domain is indicated by a gray circle. TMs 5–7 are indicated by dashed lines. **c**–**e**, AlphaFold2 model of human MARCHF6. Top (**c**) and side (**d**) views as in panels **a** and **b**, respectively, and a view into the lateral tunnel from the central cavity (**e**). Note that human MARCHF6 does not possess a structure equivalent to L8/9b in yeast (position is marked by a gray circle).

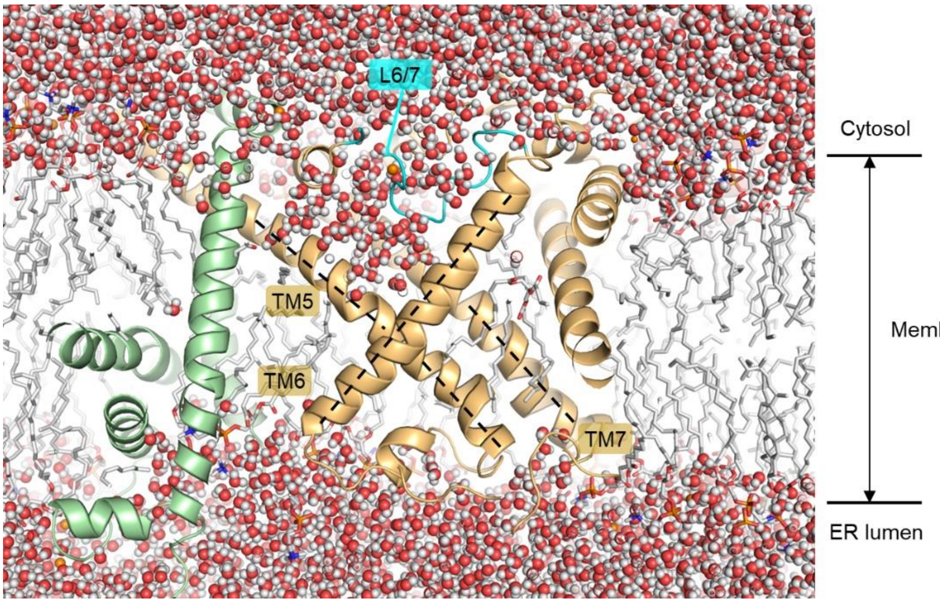

Supplementary Figure 6. Water molecules in the lateral tunnel of Doa10 in MD simulations.
An example snapshot of the all-atom MD simulation of Doa10 in a model lipid bilayer. Shown is a cross-sectional side view into the lateral tunnel of Doa10 from the central cavity. Doa10 and lipids are shown in ribbon and stick representations, respectively. TMs 5 to 7 are indicated by dashed lines. The L6/7 loop is shown in cyan. Water molecules are represented as red/gray spheres. Note that many water molecules penetrate the space between the TM5-TM6 wedge and L6/7 loop.

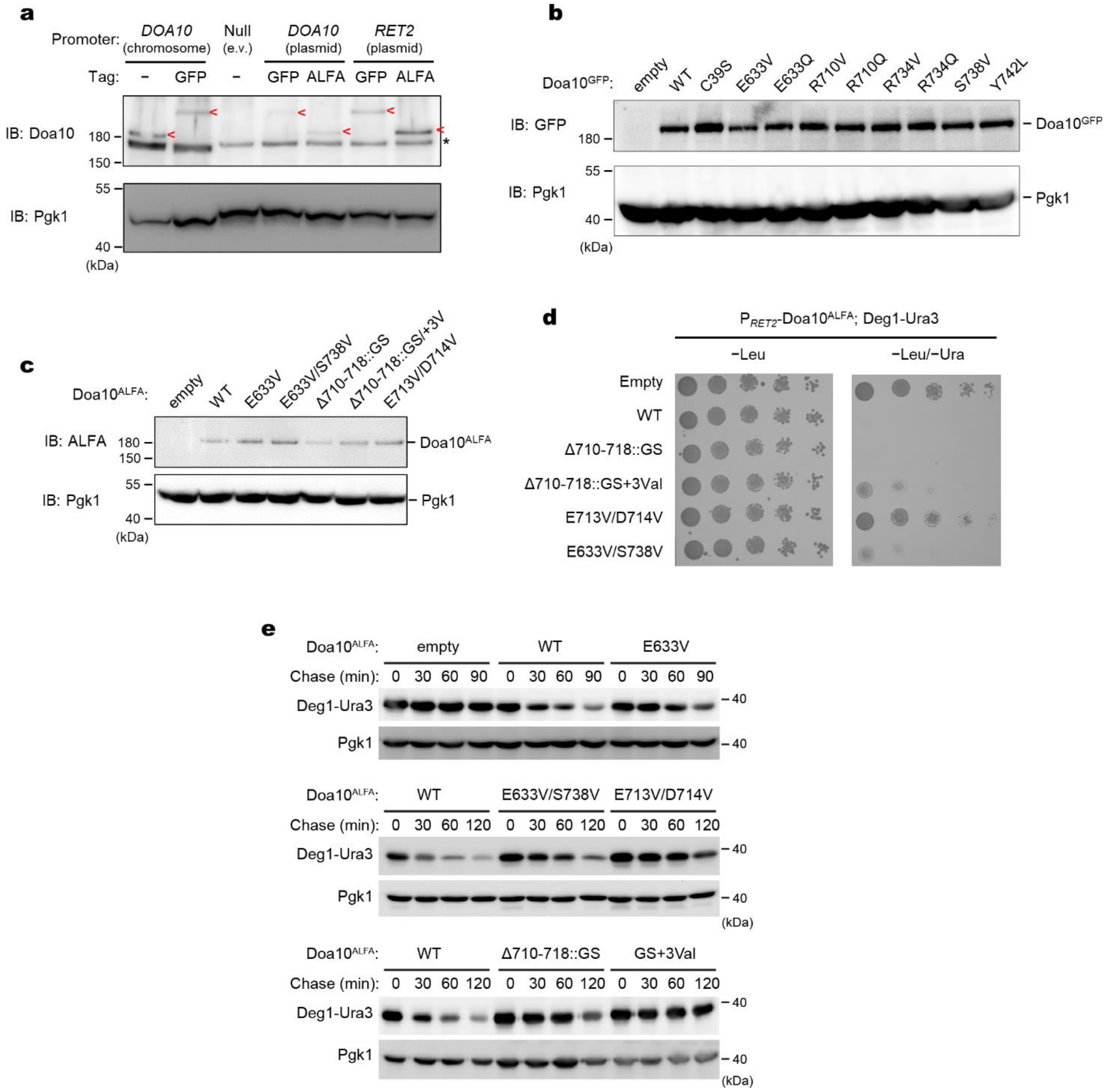

Supplementary Figure 7. Effects of Doa10 mutations on Deg1-Ura3 degradation.
a, Comparison of the endogenous and exogenous expression levels of Doa10 with either a C-terminal GFP-tag or ALFA-tag. Protein levels were measured by anti-Doa10 immunoblotting. 3-phosphoglycerate kinase (Pgk1) was used for a loading control. **b** and **c**, Expression levels of WT Doa10 and indicated mutants with a C-terminal GFP-tag (panel b) or with a C-terminal ALFA tag (panel c) was measured by immunoblotting. All the variants were expressed from a Doa10 promoter in a CEN/ARS plasmid. Pgk1 was used for a loading control. **d**, Yeast growth inhibition assay was performed as in Fig. 4e, but expressing Doa10 under the *RET2* promoter. Note that a higher expression level of Doa10 under the *RET2* promoter compared to the one under the endogenous Doa10 promoter (panel a) produces an overall stronger growth inhibition. **e**, Cycloheximide chase analysis of Deg1-Ura3 with indicated Doa10 mutants. Deg1-Ura3 levels were detected by anti-Strep-tag immunoblotting. Pgk1 was used for a loading control. See Fig. 4f for mean±s.e.m. from three independent experiments. Data in **a** and **e** are representative of three independent experiments. Data in **b**, **c**, and **d** are representative of two independent experiments.

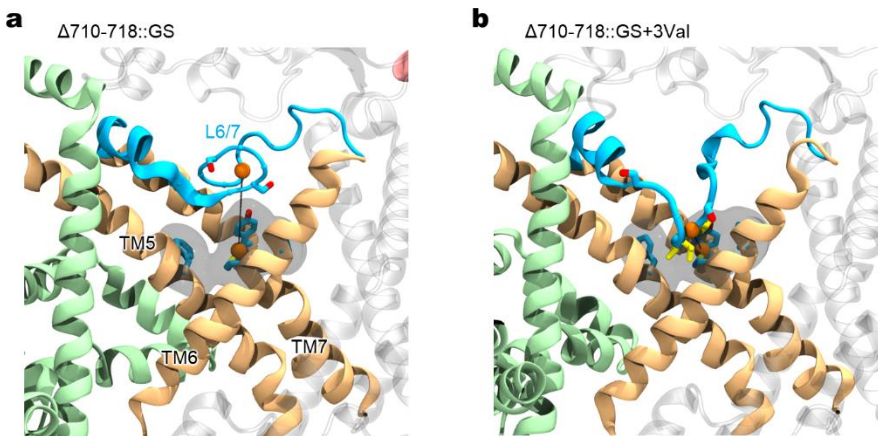

Supplementary Figure 8. Example snapshots of MD simulations on Doa10 L6/7 mutants.
a,b, As in Fig. 4 g–i, but showing example snapshots of MD simulations for the Δ710-718::GS (**a**) and Δ710-718::GS+3Val (**b**) mutant Doa10. The separation distances are 12.7 Å, and 7.7 Å for Δ710-718::GS and Δ710-718::GS+3Val, respectively. The mutated valine residues are rendered in yellow.

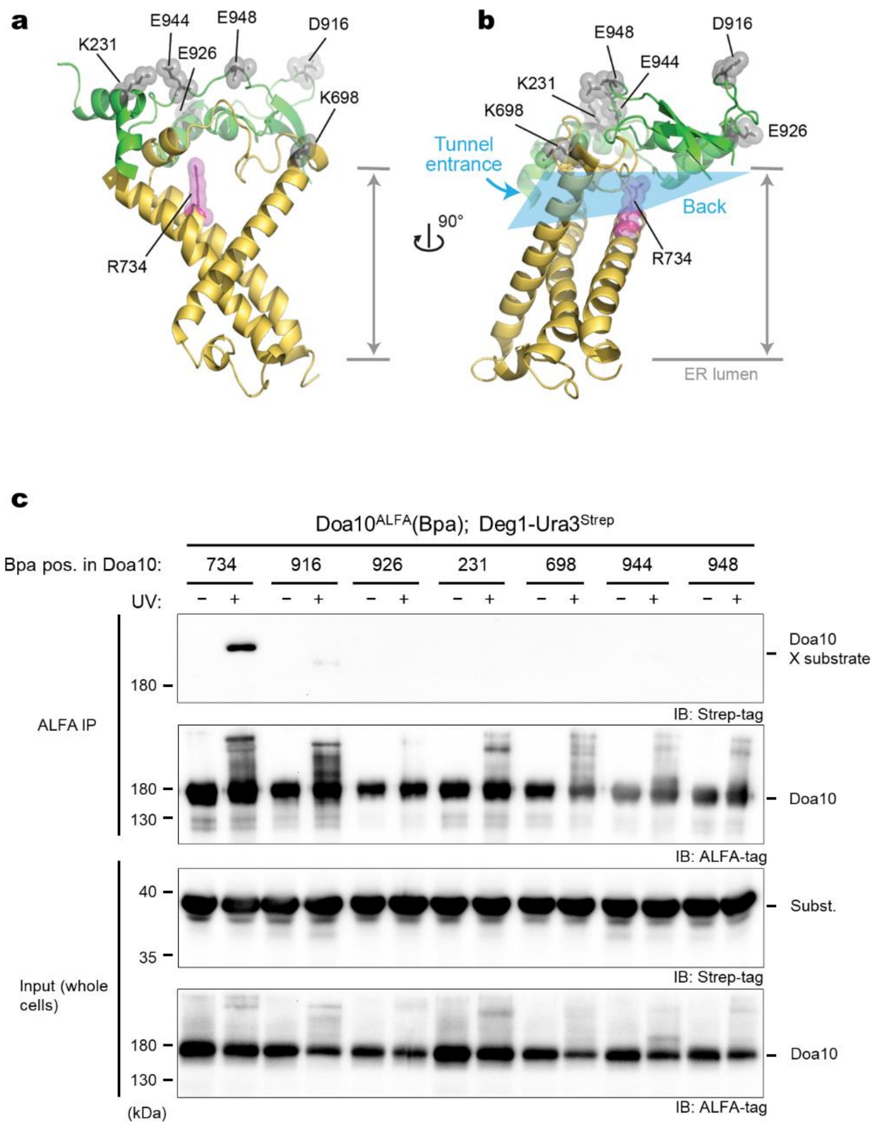

Supplementary Figure 9. Observed crosslinking between Deg1 and the Doa10 tunnel is not due to random collisions.

As in Fig. 5c–e, but showing additional Bpa sites exposed to the cytosol in the middle domain of Doa10. Panels **a** and **b** show positions of Bpa sites (see Fig. 5d,e for comparison) tested in panel **c** for UV photocrosslinking between Deg1 and Doa10. Note that position 734 is located inside the tunnel whereas all the other positions face the cytosol. Data in **c** are representative of two independent experiments.

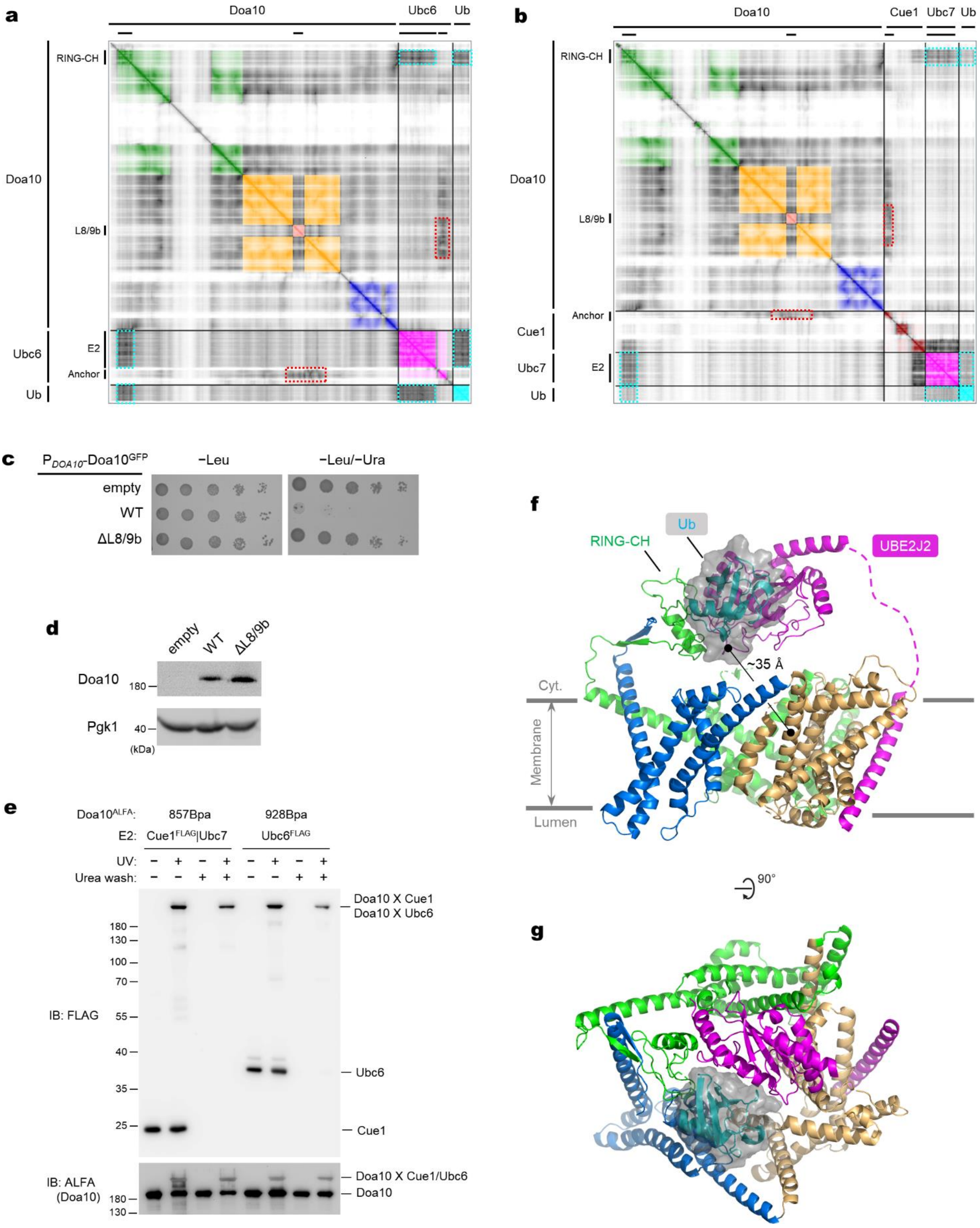

Supplementary Figure 10. Interactions between Doa10 and E2s.
(see next page for legend)

Supplementary Figure 10. Interactions between Doa10 and E2s.

a, Predicted Aligned Error (PAE) matrix for the AlphaFold2 model of the Doa10–Ubc6–Ub complex. Regions are colored according to the domain color scheme in Fig. 6 (only regions with >50 pLDDT score are in color). Dashed boxes in cyan indicate the regions corresponding to contacts between the RING-CH, E2 domain, and Ub. Dashed boxes in red indicate the contacts between the TM anchor of Ubc6 and Doa10. **b**, As in **a**, but for AlphaFold2 model for Doa10–Cue1–Ubc7–Ub. **c**, Yeast growth inhibition assay comparing the activities of WT and ΔL8/9b Doa10. Doa10 with a C-terminal GFP-tag were expressed from a *DOA10* promoter in a CEN/ARS plasmid. **d**, Expression levels of WT and ΔL8/9b Doa10 were measured by anti-GFP immunoblotting. Pgk1 was used as a loading control. **e**, Verification of covalent, UV-dependent crosslink adducts. Where indicated, ALFA nanobody beads were additionally washed with 6 M urea during immunoprecipitation to dissociate non-covalently associated proteins. **f** and **g**, As in Fig. 7a,b, but showing an AlphaFold2 model of a complex of human MARCH6, UBE2J2, and Ub. Panels in **f** and **g** show side and cytosolic views, respectively. Data in **c**, **d**, **e** are representative of two independent experiments.

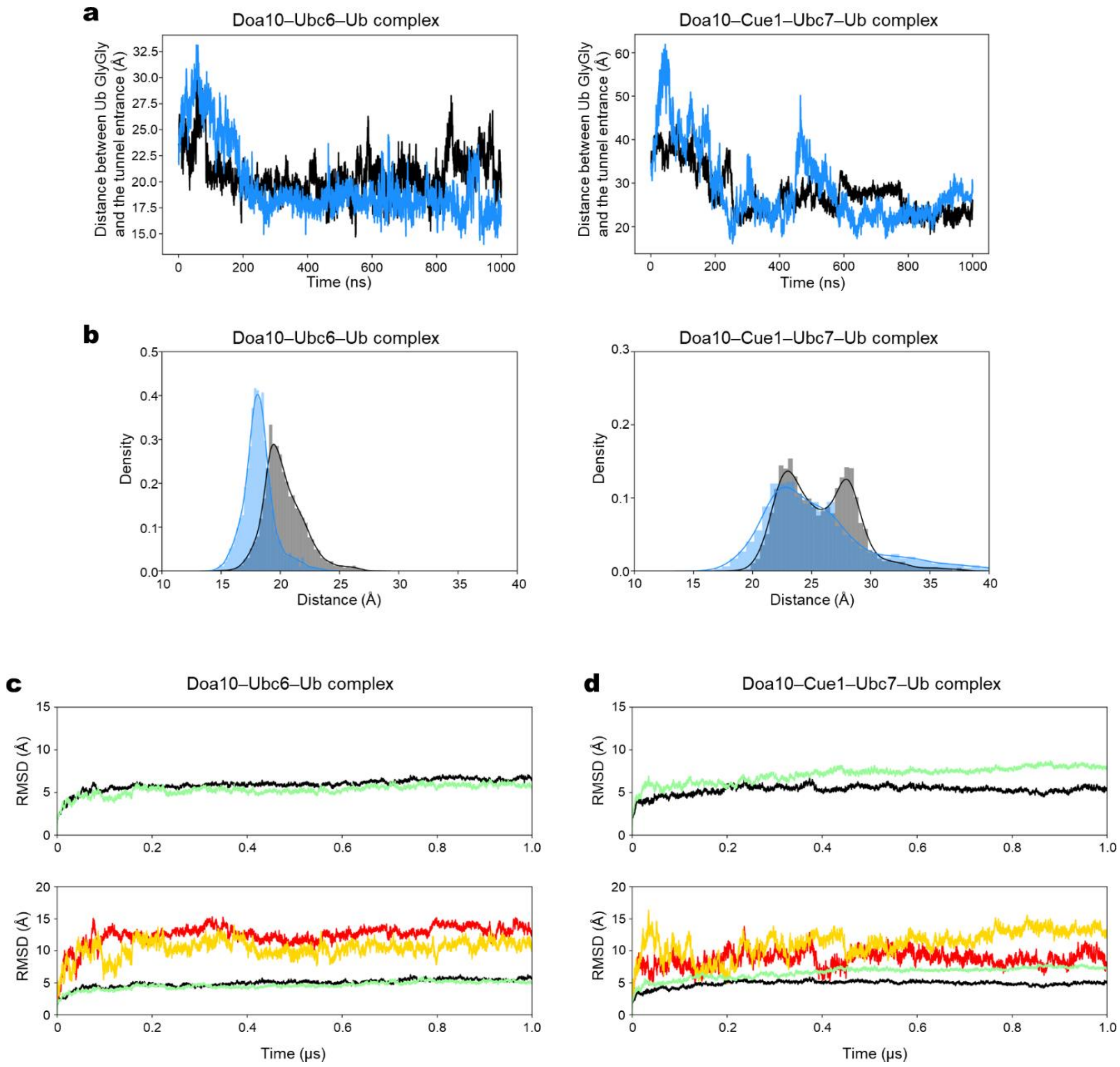

Supplementary Figure 11. Conformational flexibility of the RING-CH domain of Doa10 in complex with E2.

a, AlphaFold2 models of the Doa10–Ubc6–Ub and Doa10–Cue1–Ubc7–Ub complexes were subjected to 1-μs all-atom MD simulations in a model lipid membrane. The distance between the double glycine tail (geometric center of the Gly75 and Gly76 Cα atoms) of Ub and the tunnel entrance of Doa10 (geometric center of Leu634 and Y687 Cα atoms) was calculated over the simulation duration. Black and blue traces show two independent MD runs (replicas 1 and 2, respectively). **b**, As in a, but distance measurements after the initial 200-ns stabilization phase were plotted in histograms. Black and blue plots indicate two replicas. **c** and **d**, As in Supplementary Fig. 4b,c, but showing root-mean-square deviation (RMSD) calculated for all Cα atoms in the Doa10 models (upper panels; black and light greens are replicas 1 and 2, respectively) or calculated for the RING-CH domain (residues 30 to 118) or the membrane domain (residues 119 to 240, 463 to 1052, and 1095 to 1318) of Doa10 separately (lower panels; red and orange are for RING-CH in replicas 1 and 2, respectively. Black and light greens are the membrane domain in replicas 1 and 2, respectively). RMSD values are with respect to the initial structural model.

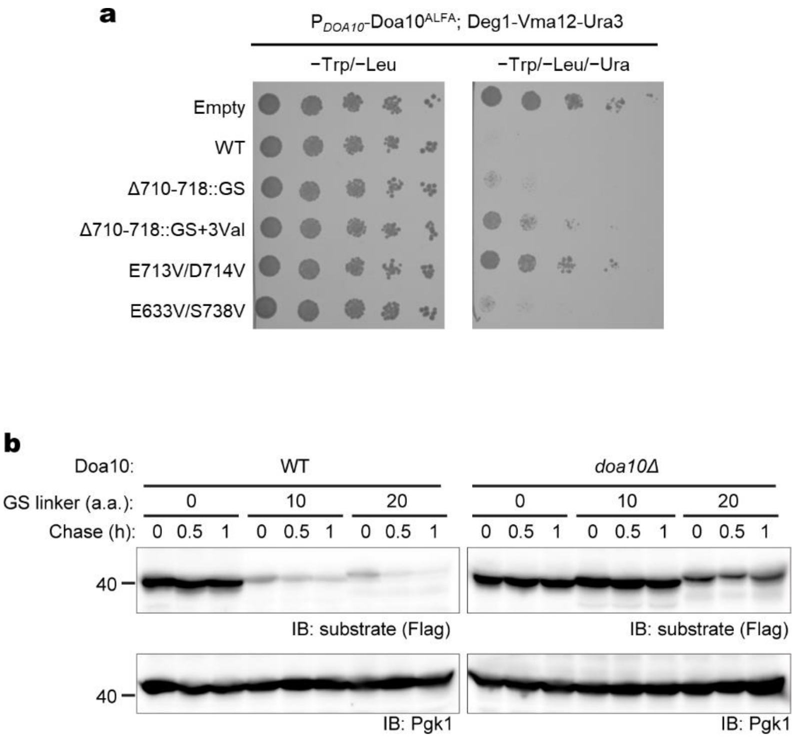

Supplementary Figure 12. Doa10-dependent degradation of Deg1-Vma12-Ura3.
a, Yeast growth inhibition assay with Deg1-Vma12-Ura3 and indicated Doa10 mutants. Deg1-Vma12-Ura3 was expressed under the *MET25* promoter, and all Doa10 mutants were expressed from the *DOA10* promoter. **b**, Cycloheximide chase analysis of Deg1$_{1\text{-}35}$-Vma12$_{\Delta 132}$-Ura3 with different lengths of the GS linker between Deg1$_{1\text{-}35}$ and Vma12$_{\Delta 132}$. Deg1 substrates were detected by anti-FLAG-tag immunoblotting (a FLAG-tag is attached to the C-terminus of Ura3). Pgk1 was used for loading control. See Fig. 8d for mean±s.e.m. from three independent experiments. Data in **a** are representative of two independent experiments. Data in **b** are representative of three independent experiments.

Table S1. Cryo-EM data collection, refinement and validation statistics.

	**Doa10 from *S. cerevisiae*** (PDB:8TQM, EMDB: 41508)
**Data collection and processing**	
Microscope	FEI Titan Krios G2
Magnification	64,000x
Voltage (kV)	300
Electron exposure (e^{-}/Å^2)	50
Defocus range (μm)	−0.7 to −2.4
Pixel size (Å)	0.91
Symmetry imposed	*C*1
Initial particle images (no.)	541,394
Final particle images (no.)	324,019
Map resolution (Å)	3.2
FSC threshold	0.143
Map resolution range (Å)	2.72 (highest), 7.15 (75% percentile)
**Refinement**	
Initial model used	De novo + AlphaFold2 model
Model resolution (Å)	3.3
FSC threshold	0.5
Map sharpening *B* factor (Å^2)	112
Model composition	
Non-hydrogen atoms	7,231
Protein residues	843
Ligands	7
B factors (Å^2)	
Protein	34.10
Ligand	12.05
R.m.s. deviations	
Bond lengths (Å)	0.005
Bond angles (°)	1.055
**Validation**	
MolProbity score	1.70
Clashscore	12.0
Poor rotamers (%)	0
Ramachandran plot	
Favored (%)	97.47
Allowed (%)	2.53
Disallowed (%)	0
CaBLAM outliers (%)	0.73

Table S2. List of yeast strains

Name	Genotype	Reference
MHY4086	*MATa his3-Δ200 ura3-52 trp1-Δ63 leu2-3112 lys2-801::LYS2::*Deg1-Ura3 *doa10Δ::HphMX4*	Stuerner et al., 2012
JY103	*MAT***a** *his3-Δ200 ura3-52 trp1-Δ63 leu2-Δ1 lys2-801 ADE2*	Laney et al., 2006
MHY10818	*MATα his3-Δ200 leu2-Δ1 ura3-52 lys2-801 trp1-Δ63 doa10Δ::hphMX4*	Mehrtash et al., 2022
BY4741	*MATa his3-1, leu2-0, met15-0, ura3-0*	Horizon Discovery
ySI-118	BY4741 *DOA10*-TEV-GFP::*natMX*	This study
ySI-154	BY4741 *leu2*::P_{GAL1}-Doa10-TEV-GFP::*natMX*	This study
ySI-167	BY4741 *doa10Δ*::*natMX*	This study
ySI-266	MHY10818 *cue1Δ*::*natMX*	This study
yKW-283	ySI-167 *leu2*::P_{PGK1}-Deg1-Ura3-2xStrep::*hphMX4*	This study
yYC-307	MHY10818 *ubc6Δ*::*natMX*	This study
yAT-055	ySI-167 *leu2*::P_{TEF1}- mScarlet(i3)-2xStrep-Sbh2::*hphMX4*)	This study
yAT-057	ySI-167 *leu2*::P_{TEF1}- mScarlet(i3)-2xStrep-Pex15Δ30::*hphMX4*)	This study

Table S3. List of plasmids

Name	Description	Reference
pYTK001 to 096	Original MoClo YTK parts	Lee et al., 2015
pYTK-e102	*LEU2* integration vector containing a hygromycin marker (assembled from pYTK008, pYTK047, pYTK073, pYTK079, pYTK087, pYTK090, and pYTK093)	This study
pYTK-e205	MoClo YTK part (type 4a) for 2x-Strep (Amino acid sequence: SGWSHPQFEKGGGSGGGSGGSAWSHPQFEK*)	This study
pYTK-e111	CEN/ARS plasmid containing a Ura3 marker (assembled from pYTK008, pYTK047, pYTK073, pYTK074, pYTK081, and pYTK084)	This study
pYTK-e112	CEN/ARS plasmid containing a Leu2 marker (assembled from pYTK008, pYTK047, pYTK073, pYTK075, pYTK081, and pYTK084)	This study
pDoa10-Doa10-split1	Split pYTK-e112 (CEN/ARS)-P_{DOA10}-Doa10(1-608)	This study
pRET2-Doa10-split1	Split pYTK-e112 (CEN/ARS)-P_{RET2}-Doa10(1-608)	This study
pTDH3-Doa10-split1	Split pYTK-e112 (CEN/ARS)-P_{TDH3}-Doa10(1-608)	This study
Doa10–GFP-split2	Split pYTK-e112 (Leu2 marker)-Doa10(608-1319)-TEV-GFP (Amino acid sequence of the tag: GTGSGTGENLYFQGTASGGGSKGEELFTGVVPILVELDGDVNG HKFSVSGEGEGDATYGKLTLKFICTTGKLPVPWPTLVTTFGYGV QCFARYPDHMKQHDFFKSAMPEGYVQERTIFFKDDGNYKTRAE VKFEGDTLVNRIELKGIDFKEDGNILGHKLEYNYNSHNVYIMADK QKNGIKVNFKIRHNIEDGSVQLADHYQQNTPIGDGPVLLPDNHYL STQSALSKDPNEKRDHMVLLEFVTAAGITHGMDELYKVDLDK*)	This study
Doa10-ALFA-split2	Split pYTK-e112 (Leu2 marker)-Doa10(608-1319)-ALFA (Amino acid sequence of the tag: GTSRLEEELRRRLTE*)	This study
pKW043	MoClo YTK part (type 3a) for Deg1	This study
pKW050	MoClo YTK part (type 3b) for Ura3	This study
pSI015	MoClo YTK part (type 3b) for Sbh2	This study
pKW022	MoClo YTK part (type 3b) for Pex15Δ30	This study
pKW070	pYTK-e111-P_{TDH3}-Deg1-Ura3-2xStrep. Assembled from pYTK-e111, pYTK009 (P_{TDH3}), pKW043 (pYTK001-Deg1), pKW050 (pYTK001-Ura3), pYTK001-e205 (2xStrep), pYTK061 (tENO1)	This study
pKW155	pYTK-e102- P_{PGK1}-Deg1-Ura3-2xstrep. Assembled from pYTK-e102, pYTK011 (P_{PGK1}), pKW043 (pYTK001-Deg1), pKW050 (pYTK001-Ura3), pYTK001-e205 (2xStrep), pYTK061 (tENO1)	This study
pKW214	MoClo YTK part (type 3) for Ura3	This study
pKW216	MoClo YTK part (type 4a) for 2xStrep-CL1	This study
pKW238	MoClo YTK part (type 3a) for mCherry-2xstrep	This study
pKW241	MoClo YTK part (type 3a) for mScarlet(i3)-2xstrep	This study
pKW265	pYTK-e111-P_{GAL1}-Ura3-2xStrep-CL1. Assembled from pYTK-e111, pYTK030 (P_{GAL1}), pKW214 (pYTK001-Ura3), pKW216 (pYTK001-2xStrep-CL1), pYTK061 (tENO1)	This study
pKW266	pYTK-e111-P_{GAL1}-Deg1-Ura3-2xStrep. Assembled from pYTK-e111, pYTK030 (P_{GAL1}), pKW043 (pYTK001-Deg1), pKW050 (pYTK001-Ura3), pYTK001-e205 (2xStrep), pYTK061 (tENO1)	This study
pKW267	pYTK-e111-P_{GAL1}-mCherry-2xStrep-Sbh2. Assembled from pYTK-e111, pYTK030 (P_{GAL1}), pKW238 (pYTK001-mCherry-2xstrep), pSI015 (pYTK001-Sbh2), pYTK051 (tENO1)	This study

pKW268	pYTK-e111-P_{GAL1}-mCherry-2xStrep-Pex15Δ30. Assembled from pYTK-e111, pYTK030 (P_{GAL1}), pKW238 (pYTK001-mCherry-2xstrep), pKW022 (pYTK001-Pex15Δ30), pYTK051 (tENO1)	This study
pKW276	pYTK-e102- P_{TEF1}-mScarlet(i3)-2xstrep-Sbh2. Assembled from pYTK-e102, pYTK013 (P_{TEF1}), pKW241 (mScarlet(i3)-2xstrep), pSI015 (pYTK001-Sbh2), pYTK051 (tENO1)	This study
pKW277	pYTK-e102- P_{TEF1}-mScarlet(i3)-2xstrep-Pex15Δ30. Assembled from pYTK-e102, pYTK013 (P_{TEF1}), pKW241 (mScarlet(i3)-2xstrep), pKW022 (pYTK001-Pex15Δ30), pYTK051 (tENO1)	This study
p414-Deg1-Vma12-Ura3	Plasmid expressing Deg1-Vma12-Ura3 from a *MET25* promoter	Ravid et al. 2006
pSK-B399-GFP-NAT	Template plasmid for PCR to generate a DNA fragment containing a C-terminal TEV-GFP tag and natMX (for chromosomal tagging).	Gift from the S. Klinge lab
pYC-300	pYTK-e111-P_{GAL1}-Cue1-2xFLAG :: P_{GAL1}-Ubc7-2xStrep	This study
pYC-301	pYTK-e111-P_{GAL1}-3xFLAG-6xHis-Ubc6	This study
pYC-302	pYTK-e111-P_{GAL1}-Cue1-2xFLAG :: P_{GAL1}-Ubc7-2xSPOT :: P_{GAL1}-3xFLAG-6xHis-Ubc6	This study
