# Supplementary material for: Substrate recognition mechanism of the endoplasmic reticulum-associated ubiquitin ligase Doa10
Source: Nat Commun. 2024 Mar 11;15:2182. doi: 10.1038/s41467-024-46409-2 (PMC10928120; doi:10.1038/s41467-024-46409-2)
Supplement: Supplementary file 3 — Description of Additional Supplementary Files [file 41467_2024_46409_MOESM3_ESM.docx]

**File name: Supplementary Movie 1.**

Description: MD simulation on the cryo-EM/AlphaFold2 hybrid model of Doa10. The hybrid model was placed in a model lipid bilayer, and an all-atom MD simulation was performed for a 1-µs duration. Shown is the side view along the membrane plane with Doa10 in a ribbon representation and positions of lipid headgroups indicated by red spheres. The color scheme is the same as in Fig. 1. The hybrid model includes the N-terminal RING-CH domain, for which an additional surface representation (surface in transparent gray) is used.

**File name: Supplementary Movie 2.**

Description: Zoom-in views into the lateral tunnel of WT and mutant Doa10 in MD simulations. Parts of TMs 5 to 7 that form the lateral tunnel are shown (front view). In the WT, E633V/S738V, and E713V/D714V panels, the L6/7 loop (positions 710-718) is shown in cyan licorice, whereas in the Δ710-718::GS (‘GS’) and Δ710-718::GS+3Val (‘GS+3Val’) mutants, the equivalent segment is shown in blue and green licorice, respectively. Gray spheres are used for a space-filling representation of the wedge-shaped tunnel-lining amino acids from TMs 5 to 7. Yellow residues are mutated valine.

**File name: Supplementary Data 1.**

Description: Cryo-EM/AlphaFold2 hybrid model of Doa10.
