# Supplementary material for: Substrate recognition mechanism of the endoplasmic reticulum-associated ubiquitin ligase Doa10
Source: Nat Commun. 2024 Mar 11;15:2182. doi: 10.1038/s41467-024-46409-2 (PMC10928120; doi:10.1038/s41467-024-46409-2)
Supplement: Supplementary file 7 — Reporting Summary [file 41467_2024_46409_MOESM7_ESM.pdf]

nature portfolio

Corresponding author(s): Eunyong Park

Last updated by author(s): 1/26/2024

Reporting Summary

Nature Portfolio wishes to improve the reproducibility of the work that we publish. This form provides structure for consistency and transparency in reporting. For further information on Nature Portfolio policies, see our Editorial Policies and the Editorial Policy Checklist.

Statistics

For all statistical analyses, confirm that the following items are present in the figure legend, table legend, main text, or Methods section.

n/a	Confirmed	
☐	☒	The exact sample size (*n*) for each experimental group/condition, given as a discrete number and unit of measurement
☐	☒	A statement on whether measurements were taken from distinct samples or whether the same sample was measured repeatedly
☒	☐	The statistical test(s) used AND whether they are one- or two-sided<br>*Only common tests should be described solely by name; describe more complex techniques in the Methods section.*
☒	☐	A description of all covariates tested
☒	☐	A description of any assumptions or corrections, such as tests of normality and adjustment for multiple comparisons
☐	☒	A full description of the statistical parameters including central tendency (e.g. means) or other basic estimates (e.g. regression coefficient) AND variation (e.g. standard deviation) or associated estimates of uncertainty (e.g. confidence intervals)
☐	☒	For null hypothesis testing, the test statistic (e.g. *F*, *t*, *r*) with confidence intervals, effect sizes, degrees of freedom and *P* value noted<br>*Give P values as exact values whenever suitable.*
☒	☐	For Bayesian analysis, information on the choice of priors and Markov chain Monte Carlo settings
☒	☐	For hierarchical and complex designs, identification of the appropriate level for tests and full reporting of outcomes
☒	☐	Estimates of effect sizes (e.g. Cohen's *d*, Pearson's *r*), indicating how they were calculated

Our web collection on statistics for biologists contains articles on many of the points above.

Software and code

Policy information about availability of computer code

Data collection	For cryo-EM collection: SerialEM v3.7
Data analysis	For cryo-EM and structural analysis: Warp v1.0.9, cryoSPARC v2.15.0, v3.0.0, and v4.3.1, Coot v0.9, Phenix v1.16, PyMOL v2.4, Chimera (various versions, but mainly 1.14), ChimeraX (various versions but mainly 1.5), AlphaFold2 v2.1.1<br>For MD simulations: CHARMM-GUI v3.7, NAMD v2.14, NAMD v3, VMD v1.9.4a51

For manuscripts utilizing custom algorithms or software that are central to the research but not yet described in published literature, software must be made available to editors and reviewers. We strongly encourage code deposition in a community repository (e.g. GitHub). See the Nature Portfolio guidelines for submitting code & software for further information.

Data

Policy information about availability of data

All manuscripts must include a data availability statement. This statement should provide the following information, where applicable:

- Accession codes, unique identifiers, or web links for publicly available datasets
- A description of any restrictions on data availability
- For clinical datasets or third party data, please ensure that the statement adheres to our policy

Cryo-EM maps and models are available through the Electron Microscopy Data Bank (EMDB) and Protein Data Bank (PDB) under the following accession codes, respectively: EMD-41508 and PDB ID 8TQM.

Research involving human participants, their data, or biological material

Policy information about studies with human participants or human data. See also policy information about sex, gender (identity/presentation), and sexual orientation and race, ethnicity and racism.

Reporting on sex and gender	N/A
Reporting on race, ethnicity, or other socially relevant groupings	N/A
Population characteristics	N/A
Recruitment	N/A
Ethics oversight	N/A

Note that full information on the approval of the study protocol must also be provided in the manuscript.

Field-specific reporting

Please select the one below that is the best fit for your research. If you are not sure, read the appropriate sections before making your selection.

☒ Life sciences ☐ Behavioural & social sciences ☐ Ecological, evolutionary & environmental sciences

For a reference copy of the document with all sections, see nature.com/documents/nr-reporting-summary-flat.pdf

Life sciences study design

All studies must disclose on these points even when the disclosure is negative.

Sample size	No statistical methods were used to predetermine sample size. For cryo-EM data, the used sample size was sufficient to achieve a high resolution 3D reconstruction to ensure sufficiently accurate modeling of our structures based on previous similar cryo-EM studies. For biochemical experiments and MD simulations, sample sizes were chosen based on commonly used sample sizes in the field. Generally, we repeated experiments two or three times (three times or more in case means and errors were calculated) to ensure reproducibility.
Data exclusions	No data was systematically excluded. The process of generating 3D maps from cryo-EM particles involves sorting for particles that are damaged, have weak signal, or are unlikely to refine correctly. This is a standard practice in the field and was implemented using Warp and cryoSPARC.
Replication	There was no attempt to replicate cryo-EM results because sufficient data was collected in the initial trial, as described above. All biochemical experiments (yeast growth assays, co-immunoprecipitation assays, SEC, cycloheximide chase assay, photocrosslinking) have been replicated with at least two independent experiments. The number of replications for each experiment is stated in the Figure Legends. MD simulations were also replicated twice.
Randomization	Randomization was not attempted or needed for this study, since this study did not allocate experimental groups.
Blinding	Blinding was not attempted or needed for this study because no subjective allocation was involved in the design of the experiments

Reporting for specific materials, systems and methods

We require information from authors about some types of materials, experimental systems and methods used in many studies. Here, indicate whether each material, system or method listed is relevant to your study. If you are not sure if a list item applies to your research, read the appropriate section before selecting a response.

Materials & experimental systems

n/a	Involved in the study	
☐	☒	Antibodies
☒	☐	Eukaryotic cell lines
☒	☐	Palaeontology and archaeology
☒	☐	Animals and other organisms
☒	☐	Clinical data
☒	☐	Dual use research of concern
☒	☐	Plants

Methods

n/a	Involved in the study	
☒	☐	ChIP-seq
☒	☐	Flow cytometry
☒	☐	MRI-based neuroimaging

Antibodies

Antibodies used	Rabbit polyclonal anti-Doa10 (gift from M. Hochstrasser; homemade stock, 1:1,000), Mouse monoclonal anti-GFP (Thermo Fisher Cat# MA5-15256; Lot# SH253777; 1:3,000), Rabbit Fc-fused monoclonal anti-ALFA-tag nanobody (homemade; 1:500), Rabbit Fc-fused anti-SPOT-tag nanobody (homemade; 1:500), rabbit polyclonal anti-Strep-tag (Genscript Cat# A00626; Lot# P2109021; 1:2,000), mouse monoclonal anti-FLAG-tag (Sigma Cat# F1804; Lot# SLBG5673V; 1,1000), anti-Pgk1 antiserum (gift from J. Thorner; 1:1,000). Secondary antibodies conjugated to HRP used in this study were goat anti-rabbit (Thermo #31460, 1:10,000), goat anti-mouse (Thermo #31430, 1:10,000).
Validation	All primary antibodies were validated by control immunoblots in the presence of molecular weight markers and performed as expected according to previous papers and manufacturers' websites. The anti-Doa10 was described in Stefan G. Kreft, Lin Wang, Mark Hochstrasser (2006) Journal of Biological Chemistry 281 (8), 4646-4653. The anti-Pgk1 antibody was described in Baum P, Thorner J, Honig L (1978) Proc. Natl. Acad. Sci. USA 75:4962-4966 and many other papers by J. Thorner. The anti-GFP (https://www.thermofisher.com/antibody/product/GFP-Antibody-clone-GF28R-Monoclonal/MA5-15256), anti-Strep (https://www.genscript.com/antibody/A00626-NWSHPQFEK_Antibody_pAb_Rabbit.html), and anti-FLAG (https://www.sigmaaldrich.com/US/en/product/sigma/f1804) antibodies are described on their manufacturer's websites. The anti-ALFA-tag and anti-SPOT-tag antibodies were home-made by fusion of ALFA (https://doi.org/10.1038/s41467-019-12301-7) or SPOT (also known as BC2; https://doi.org/10.1038/srep19211) nanobody to rabbit Fc and have been described previously (https://doi.org/10.1038/s41586-023-06239-6).
